# Heat Source/Sink in a Magneto-Hydrodynamic Non-Newtonian Fluid Flow in a Porous Medium: Dual Solutions

**DOI:** 10.1371/journal.pone.0162205

**Published:** 2016-09-06

**Authors:** Tasawar Hayat, Muhammad Awais, Amna Imtiaz

**Affiliations:** 1 Department of Mathematics, Quaid-i-Azam University, Islamabad, Pakistan; 2 Department of Mathematics, COMSATS Institute of Information Technology, Attock, Pakistan; Tsinghua University, CHINA

## Abstract

This communication deals with the properties of heat source/sink in a magneto-hydrodynamic flow of a non-Newtonian fluid immersed in a porous medium. Shrinking phenomenon along with the permeability of the wall is considered. Mathematical modelling is performed to convert the considered physical process into set of coupled nonlinear mathematical equations. Suitable transformations are invoked to convert the set of partial differential equations into nonlinear ordinary differential equations which are tackled numerically for the solution computations. It is noted that dual solutions for various physical parameters exist which are analyzed in detail.

## Introduction

The hydro-magnetic fluid flow problems combined with the effects of heat transport phenomenon in a porous system are one of the useful problems in the field of fluid engineering because of their rheological implication and applied germaneness. Sheeting stuff (paper, fiber and metallic sheets), glass blowing and manufacturing, casting and coating of wires, synthetic fiber, purification of molten metals from a nonmetallic inclusion due to magnetic field application, crystal growing etc. Recently various applied mathematicians and researchers have proposed that the cooling rate is critical for the products in order to enhance their quality. For-example in extracting metals from ores, the phenomenon of heat transfer is very advantageous. Therefore various recent researchers in the field from all progressed countries have been engaged in investigating different fluid mechanics problems under various flow configurations including suction/blowing phenomenon at the boundaries, magneto-hydrodynamics, internal heat generation/absorption, rotation effects, permeability of porous medium, simultaneous effects of energy and concentration process, viscous dissipations and Joule and Newtonian heating process etc. For-instance general solutions for the hydro-magnetic natural convection flow over a moving surface has been studied by Fetecau et al. [1]. The authors have presented the radiative heat transfer analysis with slip boundary conditions. Laplace transform technique has been incorporated to compute the close form solutions for the considered analysis and it is noted that the obtained solutions depends upon the slip parameter. Mehmood and Fetecau [2] presented a note on the radiative heat transfer to the flow of Sisko fluid. Pressure driven flow in an asymmetric channel is considered and the effects of non-uniform wall temperature are presented. The solutions in the analytical form are computed for the stream functions, axial velocities and pressure. Sheikoleslami et al. [3] investigation the rheology of the micropolar fluid in a channel with heat transfer effects. Additional effects of chemical reactions are studied and the solutions are computed by using the homotopy perturbation method. Effects of significant quantities including Reynolds number, micro-rotation, Peclet number etc are presented in detail. Combined effects of heat and mass transfer of a micropolar fluid in a porous channel has been analyzed by Sheikoleslami et al. [4]. Authors have utilized the differential transform method (DTM) for the solution computations. Effects of several physical quantities including coupling and spin gradient viscosity on the fluid have been analyzed in detail. Effects of heat transfer on a third grade fluid in a channel has been presented by Ellahi et al. [5]. They have discussed the rheology of an incompressible third grade over a porous wall and computed an analytical solutions for the velocity and temperature profiles. Ellahi [6] predicted the effects of MHD and temperature dependent viscosity on the flow of non-Newtonian nanofluid in a pipe. Adesanya and Falade [7] presented the thermodynamics analysis of the hydro-magnetic third grade fluid flow through a channel filled with porous medium. Authors have discussed the entropy generation and heat transfer rate across the channel. Turkyilmazoglu [8] computed the dual and triple solutions for the hydro-magnetic slip flow of non-Newtonian fluid over a shrinking wall. He targeted the exponential type solutions to analyze whether they are unique or multiple under slip conditions. Awais et al. [9] studied the combined effects of Newtonian heating, thermal diffusion and diffusion thermos on an axisymmetric non-Newtonian fluid flow. Zahid et al. [10] presented an analysis on the stagnation point flow induced by exponentially stretching wall with viscous dissipation and thermal radiation. Analytic approximate solutions for MHD boundary-layer viscoelastic fluid flow over continuously moving stretching surface has been presented by Rashidi et al. [11]. They have employed homotopy analysis method (HAM) with two auxiliary parameters to construct the solutions. Mixed convective heat transfer for MHD viscoelastic fluid flow over a porous wedge with thermal radiation has been analyzed by Rashidi et al. [12]. The non-Newtonian flow phenomenon over a wedge with porous has been investigated by the authors in details. Rashidi et al. [13] investigated the free convective heat and mass transfer for MHD fluid flow over a permeable vertical stretching sheet. Authors have also presented the combined effects of radiation and buoyancy in their analysis. Unsteady MHD free convective flow past a permeable stretching vertical surface in a nano-fluid has been studied by Freidoonimehr et al. [14]. Heat and mass transfer analysis of hydro- magnetic nanofluid flow in a rotatinf channel with slip effects has been presented by Raza et al. [15]. Authors have presented the numerical solutions for the slip flow regime.

The aim of this investigation is to extend the topic of heat transfer in a non-Newtonian fluid flow into the new direction. In current analysis we have presented the existence of dual solutions for the internal heat generation/absorption phenomenon in a non-Newtonian fluid flow. Numerical solutions are computed for the nonlinear differential system. The plot of streamlines for the Newtonian and Maxwell models are presented showing a different of rheology between the considered problems. The plots for internal heat generation/absorption, porosity, suctions/injection and Deborah number are presented showing the existence of the dual nature of solutions for the velocity and temperature profiles. A table has been constructed in order to present the numerical values of local Nusselt number for different involved physical quantities.

## Statement of Problem

Let us consider the non-linear boundary layer flow of an upper-convected Maxwell (UCM) fluid over a surface which is shrinking in its own place. The permeability of the surface is considered to work out the effects of suction and blowing. The fluid saturates the porous medium *y >0* and flow occupies the region of positive *y*– axis. Moreover the effect of applied magnetic field are considered parallel to deformation axes. The mathematical equations representing the considered physical phenomenon are given by

$$\frac{\partial u}{\partial x}+\frac{\partial u}{\partial y}=0,$$ (1)

$$\begin{array}{l} u\frac{\partial u}{\partial x}+v\frac{\partial u}{\partial y}+\lambda \left(u^{2}\frac{\partial^{2}u}{\partial x^{2}}+v^{2}\frac{\partial^{2}u}{\partial y^{2}}+2uv\frac{\partial^{2}u}{\partial x\partial y}\right)=\nu \frac{\partial^{2}u}{\partial y^{2}}-\frac{\sigma B_{0}^{2}}{\rho }(u+\lambda v\frac{\partial u}{\partial y})\\ -\frac{\nu }{K}(u+\lambda v\frac{\partial u}{\partial y}), \end{array}$$ (2)

where the u and *v* are the velocity component, (*x*,*y*) represents the coordinate system, *λ* is relaxation time, *ν* is kinematic viscosity, *σ* is electrical conductivity, *B*_0_ is magnetic field strength, *ρ* is fluid density and *K* is permeability of porous medium. The wall conditions for the present physical system are given by

$$\begin{array}{l} \;u=U_{w},\;v=V_{w},\;\text{at}\;y=0,\\ u\to 0,\;\text{as}\;y\to \infty , \end{array}$$ (3)

where *U*_*w*_ = −*cx* is the shrinking velocity of wall where *c* > 0 and *V*_*w*_ is the suction/blowing parameter. It is pointed out here that *V*_*w*_ < 0 represents suction phenomena where *V*_*w*_ > 0 corresponds to blowing situation.

The stream function *ϕ* is introduced for velocity components *u* and *v* as

$$u=\frac{\partial \phi }{\partial y}\;\text{and}\;v=-\frac{\partial \phi }{\partial x}.$$ (4)

Utilizing Eq (4) in Eq (2), we get

$$\begin{array}{l} \phi_{y}\phi_{xy}-\phi_{x}\phi_{yy}+\lambda \left({\left(\phi_{y}\right)}^{2}\phi_{xxy}+{\left(\phi_{x}\right)}^{2}\phi_{yyy}-2\phi_{y}\phi_{x}\phi_{xyy}\right)=\nu \phi_{yyy}\\ -\frac{\sigma B_{0}^{2}}{\rho }\left(\phi_{y}-\lambda \phi_{x}\phi_{yy}\right)-\frac{\nu }{K}\left(\phi_{y}-\lambda \phi_{x}\phi_{yy}\right), \end{array}$$ (5)

whereas Eq (1) is satisfied identically.

Similarly the wall conditions (Eq (3)) can be transformed as,

$$\begin{array}{l} \;\frac{\partial \phi }{\partial y}=U_{w},\;\frac{\partial \phi }{\partial x}=-V_{w},\;\text{at}\;y=0,\\ \frac{\partial \phi }{\partial y}\to 0,\;\text{as}\;y\to \infty . \end{array}$$ (6)

The stream function *ϕ* in the dimensionless form is given by

$$\phi =\sqrt{c\nu }xf(\eta )$$ (7)

where $\eta =\sqrt{\frac{c}{\nu }}y$ is the similarity variable. Making use of Eq (7), we can get the self-similar form of Eq (5) given by

$$f^{\prime \prime \prime }-{\left(f^{\prime }\right)}^{2}+ff^{\prime \prime }+\beta \left(2ff^{\prime }f^{\prime \prime }-f^{2}f^{\prime \prime \prime }\right)+M^{2}\beta ff^{\prime \prime }-M^{2}f^{\prime }-K_{1}f^{\prime }+K_{1}\beta ff^{\prime \prime }=0,$$ (8)

where *β* = *λc* represents the Deborah number in terms of relaxation time, $M^{2}=\frac{\sigma B_{0}^{2}}{c\rho }$ is the magnetic parameter, $S=-V_{w}/\sqrt{\nu c}$ is the suction/blowing parameter where *S* > 0 means wall mass suction and *S* < 0 means wall mass injection and $K_{1}=\frac{\nu }{Kc}$ is the porosity parameter respectively.

The wall conditions in dimensionless form are

$$f(0)=S,\;f^{\prime }(0)=-1,f^{\prime }(\infty )=0$$ (9)

where prime represents differentiation with respect to *η*.

## Heat generation/absorption phenomenon

The energy equations representing the temperature distribution in the rheological system (when internal heat generation/absorption effect are present) takes the form

$$u\frac{\partial T}{\partial x}+v\frac{\partial T}{\partial y}=\alpha_{m}\frac{\partial^{2}T}{\partial y^{2}}+\frac{Q}{\rho c_{p}}(T-T_{\infty }),$$ (10)

where temperature *T*, thermal diffusivity $\alpha_{m}=\frac{k}{\rho c_{p}},$ thermal conductivity *k*, specific heat *c*_*p*_, internal heat generation/absorption coefficient *Q*, free stream temperature *T*_∞_ are the different thermal and rheological quantities. The appropriate wall conditions for temperature distribution are

$$\begin{array}{l} T=T_{w}\;\text{as}\;y=0,\\ T=T_{\infty }\;\text{as}\;y\to \infty , \end{array}$$ (11)

where *T*_*w*_ is the constant wall temperature. The dimensionless "*θ*" is introduced through the following equation

$$\theta (\eta )=\frac{T-T_{\infty }}{T_{w}-T_{\infty }}.$$ (12)

Making use of the Eq (7) and Eq (12) in Eq (10), we get

$$\theta^{\prime \prime }+\mathrm{Pr}f\theta^{\prime }+\mathrm{Pr}\lambda_{1}\theta =0,$$ (13)

where $\mathrm{Pr}=\frac{\upsilon }{\alpha_{m}}$ represents the Prandtl number and $\lambda_{1}=\frac{Q}{c\rho c_{p}}$ is the internal heat generation/absorption parameter. Moreover the dimensionless thermal conditions are given by

θ(0)=1,θ(∞)=0. (14)

The local Nusselt number *Nu*_*x*_ is the physical quantity of interest for the readers. It is defined as

$$Nu_{x}=\frac{xq_{w}}{k(T_{w}-T_{\infty })},$$ (15)

where *q*_*w*_ (the wall heat flux) is defined as

$$q_{w}=-k{\left(\frac{\partial T}{\partial y}\right)}_{y=0}.$$ (16)

In dimensionless form we can write the above expression as

$$Nu_{x}/Re_{x}^{1/2}=-\theta^{\prime }(0).$$ (17)

## Numerical Computations

The nonlinear coupled differential Eqs (8 and (13)) along with conditions (Eq (9) and Eq (14)) are computed numerically for solutions and analyzed in detail in order to discuss the effects of different pertinent parameters including Deborah number, internal heat generation/absorption phenomenon, wall mass transfer, porosity and magnetic field etc. An efficient approach namely shooting method (combination of Runge-Kutta fourth-order algorithm and Newtons' method) is employed. The coupled non-linear equations (Eq (8) and Eq (13)) are initially converted into initial value problem (IVP) by setting

$$\begin{array}{l} f^{\prime }=p,\;p^{\prime }=q,\;q^{\prime }=\frac{p^{2}-fq-2\beta fpq-\beta M^{2}fq-\beta K_{1}fq+M^{2}p+K_{1}p}{1-\beta f^{2}},\\ \theta^{\prime }=z,\;\theta_{1}^{\prime }=-\mathrm{Pr}\lambda_{1}\theta -\mathrm{Pr}fz, \end{array}$$ (18)

with the wall conditions,

f(0)=S,p(0)=−1,θ(0)=1, (19)

whereas the value of *q*(0) and *z*(0) are required to solve the IVPS (Eqs (18 and 19). The appropriate initial value for *q*(0) and *z*(0) are selected and the shooting algorithm is employed to approximate the final answers up to the desired accuracy with in a tolerance level of 10^−5^. If needed we can provide the complete numerical code to the research community as a goodwill gesture so that anyone can venture further.

## Results and Discussion

**Fig 1:**
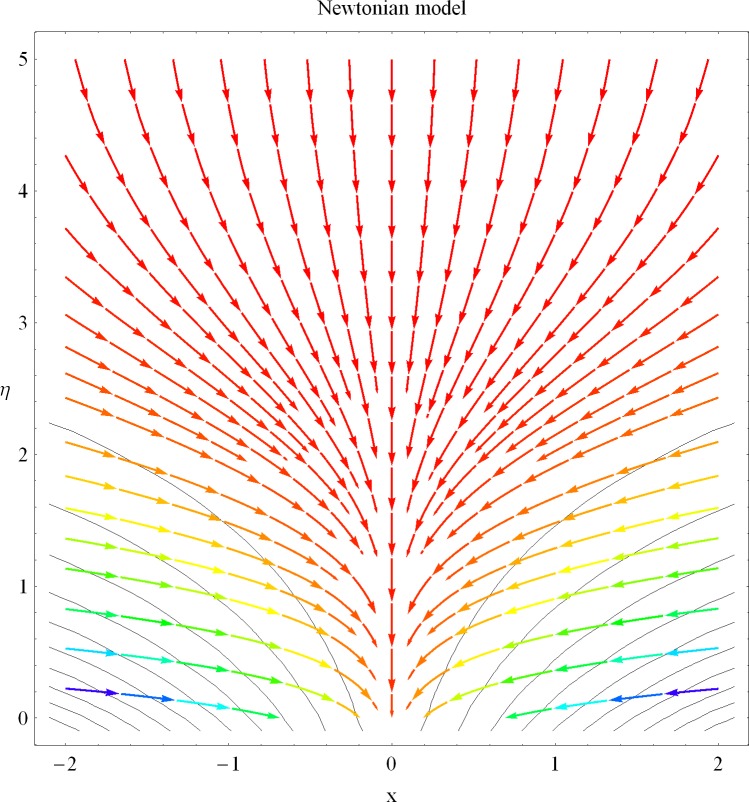
Streamlines for Newtonian model.

**Fig 2:**
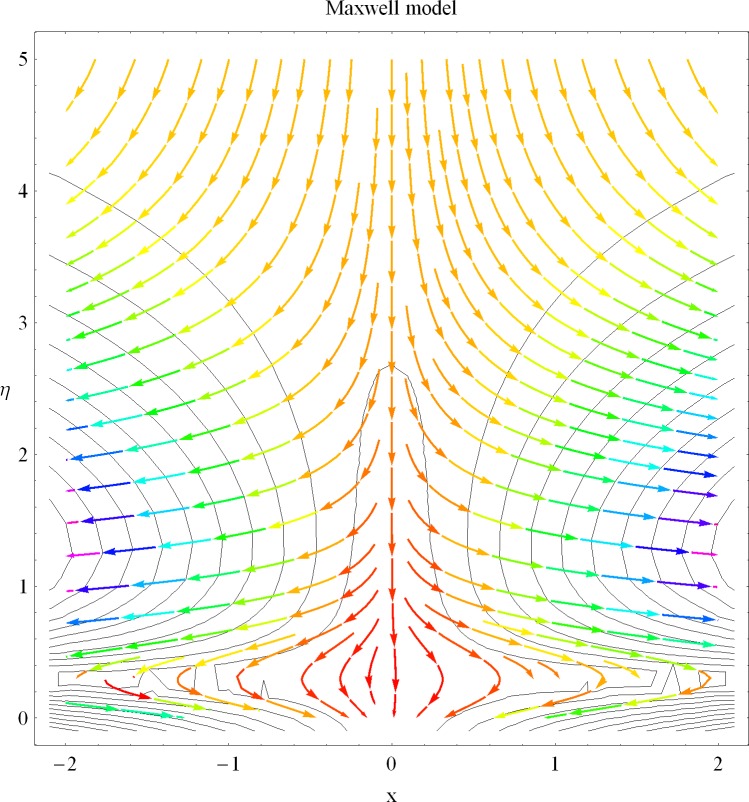
Streamlines for Maxwell model.

**Fig 3:**
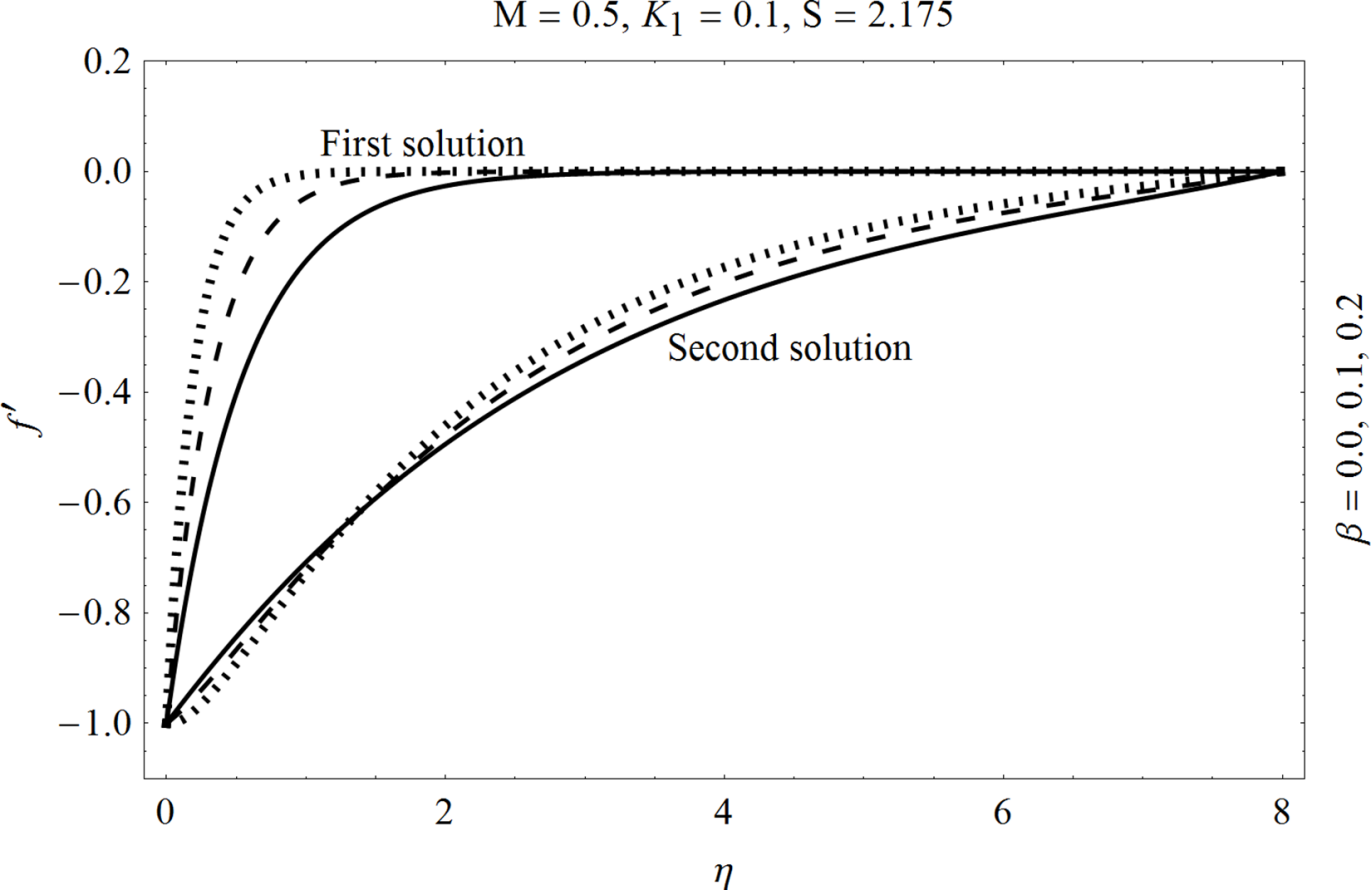
Effects of Deborah number *β* on *f*′.

**Fig 4:**
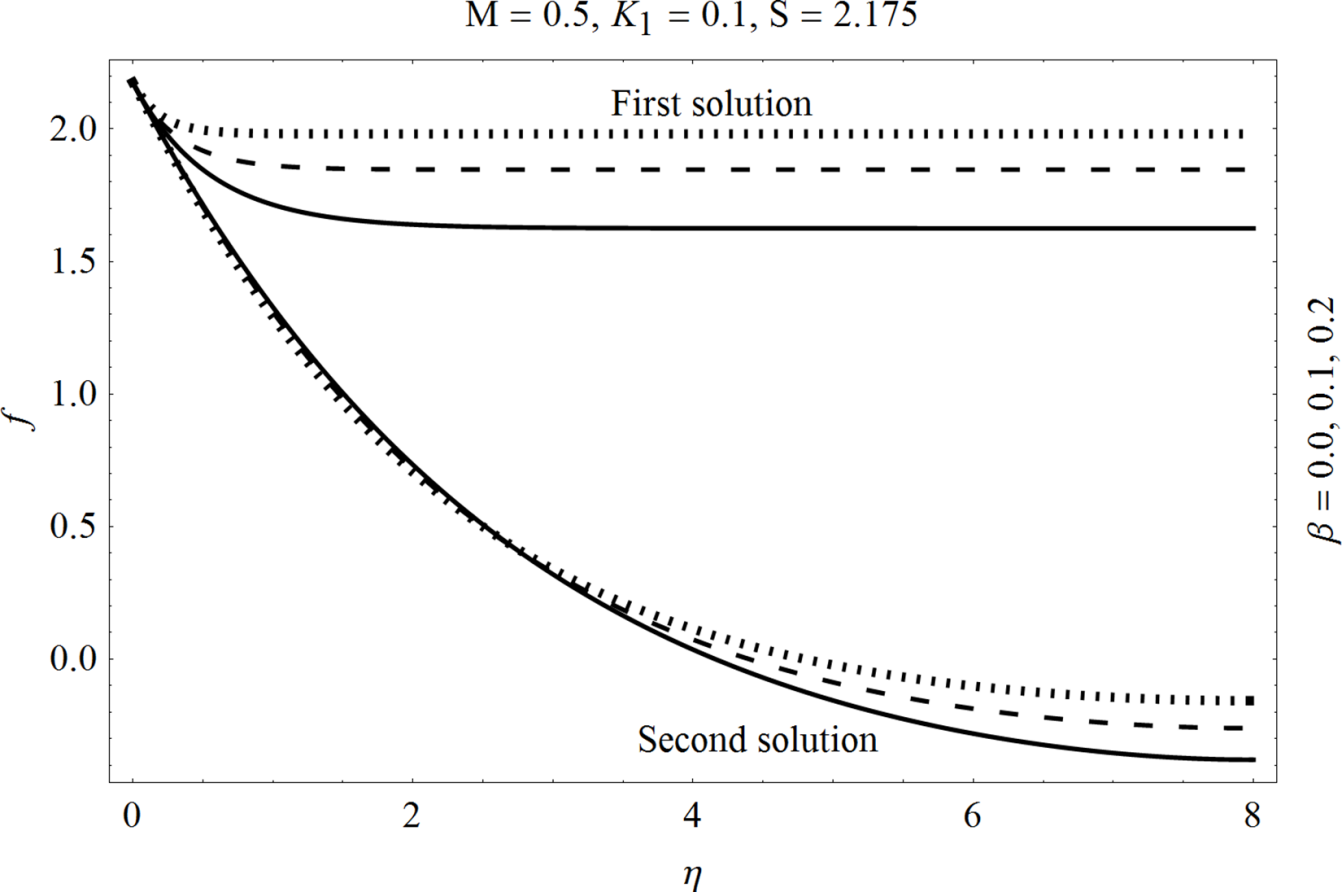
Effects of Deborah number *β* on *f*.

**Fig 5:**
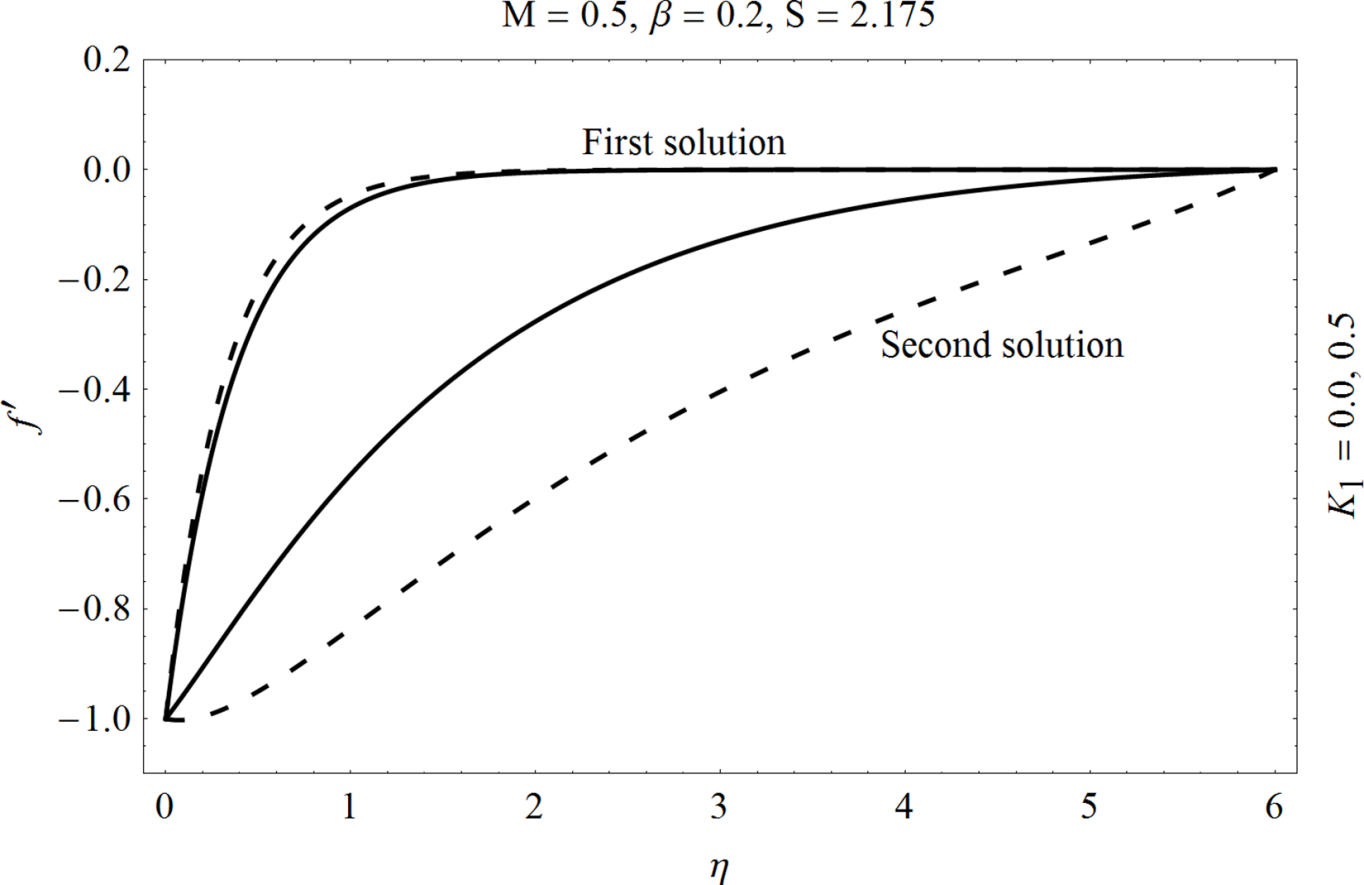
Effects of porosity parameter *K*_*1*_ on *f*′.

**Fig 6:**
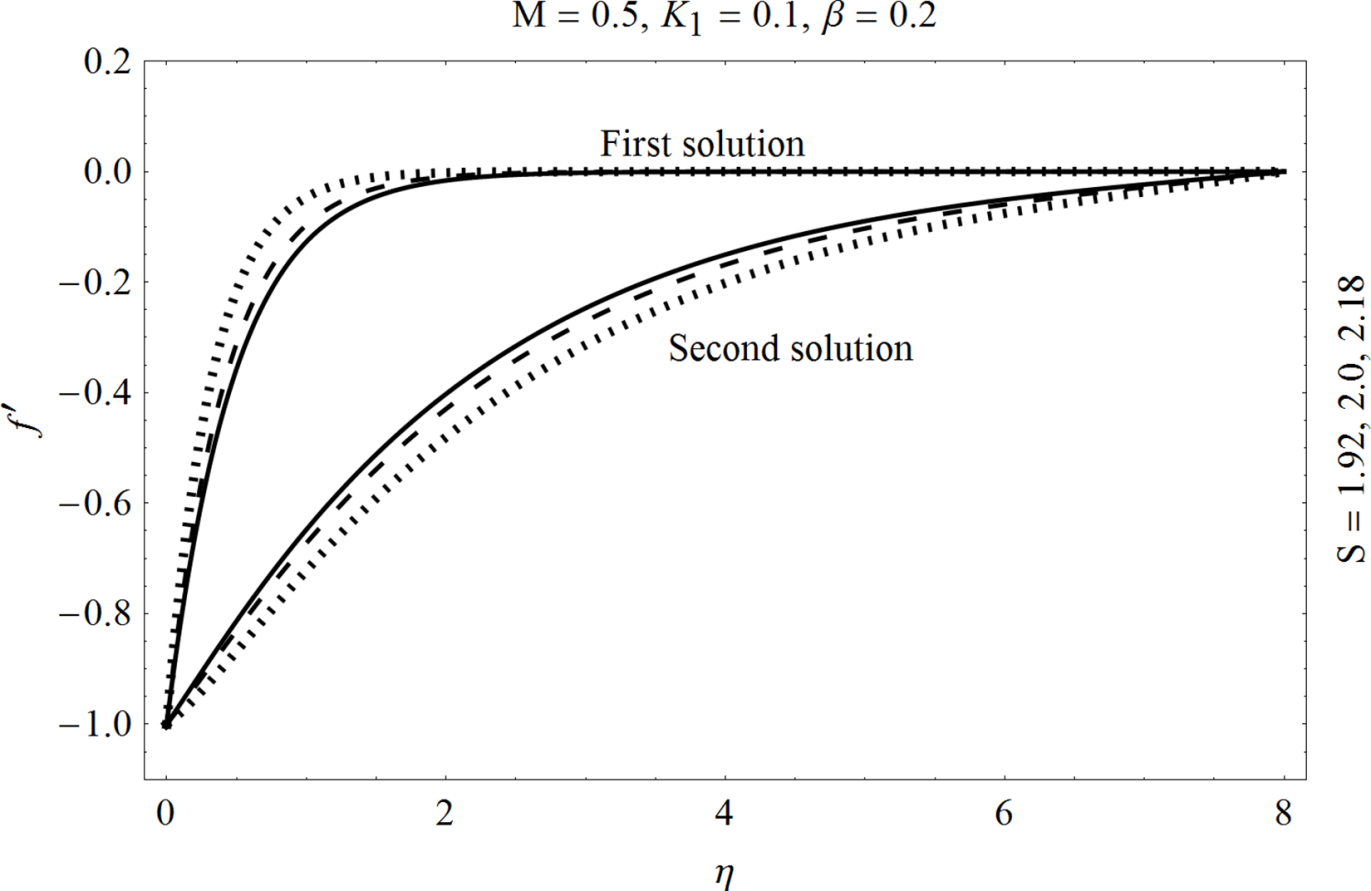
Effects of suction/injection S on *f*′.

**Fig 7:**
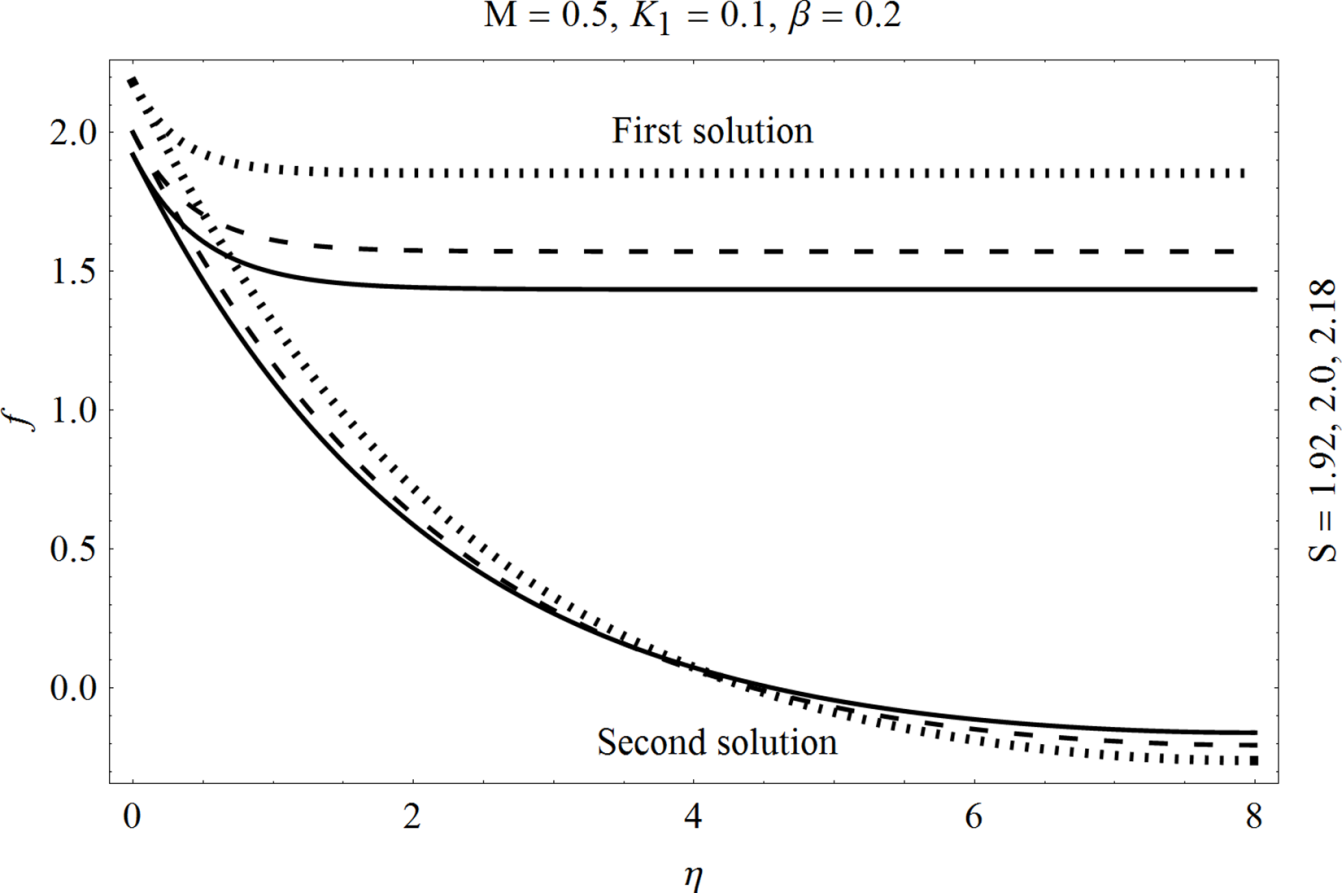
Effects of suction/injection S on *f*.

**Fig 8:**
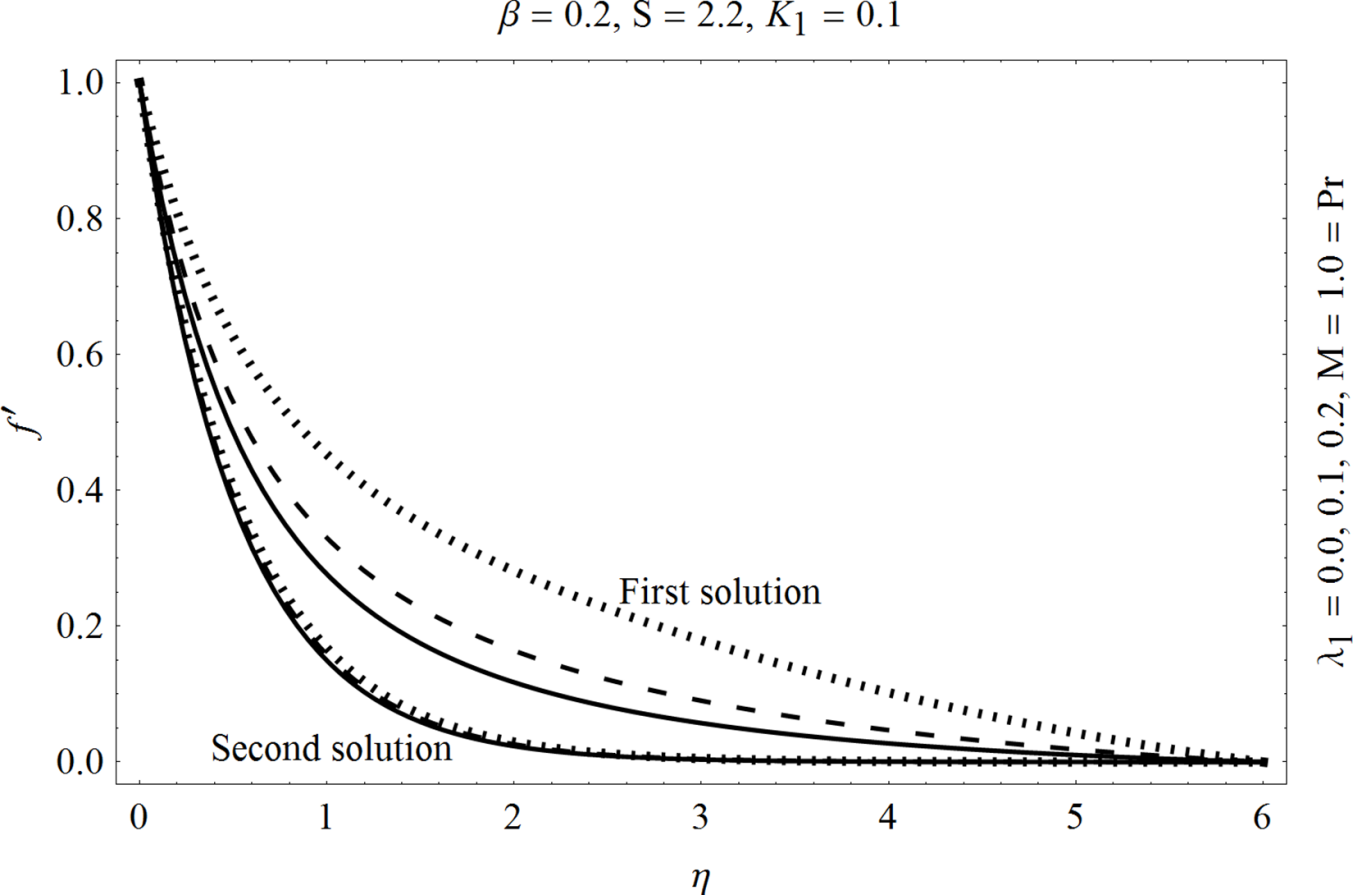
Effects of heat gen. *λ*_1_> 0 on *f*′.

**Fig 9:**
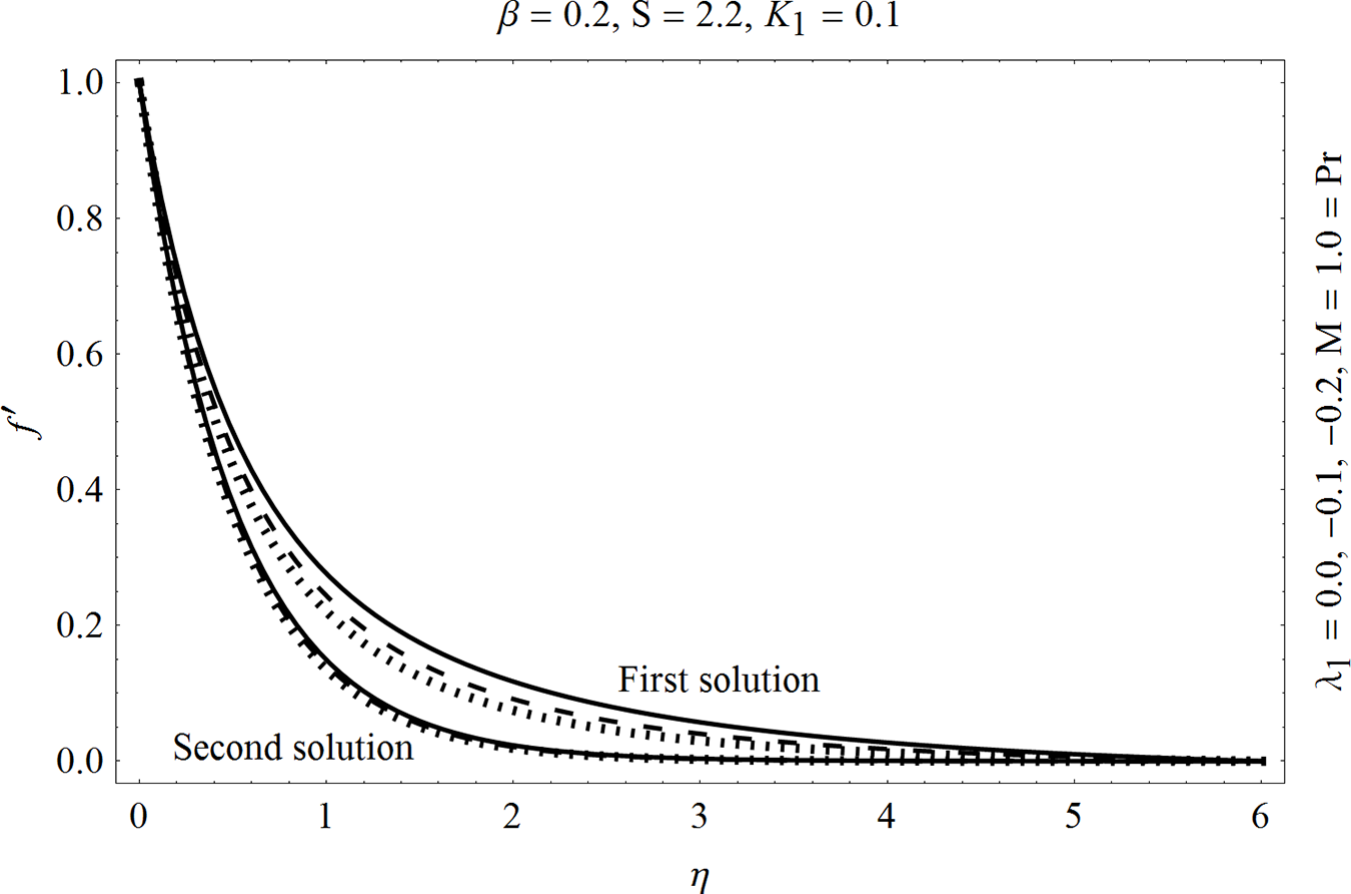
Effects of heat absorption *λ*_1_< 0 on *f*′.

In the sections we have presented several graphical illustrations and numerical values showing the rheology of the considered analysis. For this purpose we have prepared Figs 1–9 and Table 1 in order to presented the effects of Deborah number, porosity parameter, suction/injection quantity, heat generation and absorption and local Nusselt number etc. Figs 1 and 2 elucidate the stream line behavior for Newtonian and Maxwell models. It is observed that in present analysis streamline for Newtonian and Maxwell models are quite different form each other. Figs 3 and 4 depict the influence of Deborah number *β* on the velocities *f*′ and *f* .respectively. It is noticed that the dual solutions exist for Deborah number *β*. Moreover, it is also evident that boundary layer thickness in both solution decreases for larger values of *β*. with an increase in *η*. Since for larger Deborah number, viscous effects increase which retards the flow and hence the momentum boundary layer will be thinner. Moreover it is also clear from Figs 1 and 2 that oscillations and crossover are observed in second solution profile since the Deborah is the ratio of relaxation time (time for a material to adjust to apply stresses or deformations) and the characteristic time of an experiment (or a computer simulation giving the response of a material). Since Deborah number *β* defines the difference between the solids and liquids (or fluids). The material show fluid like behavior for smaller Deborah number where as for large value of Deborah number, the material behaves like viscoelastic solid (e.g. rubber, jelly, polymers etc.). Therefore it is quite obvious from the present analysis that velocity field shows deceleration for larger Deborah number. Fig 5 displays the effects of porosity parameter *K*_*1*_ on *f*′. It is observed that dual solutions exist for different values *K*_*1*_. The first solutions shows small variations in the velocity which as second solutions presents quite significant change. Moreover under the applications of constant magnetic field M due to the Lorentz force the momentum boundary layer decreases and velocity field retards which is shown in the second solution. Therefore we can say that the second solutions is dominant when compared with the first solution. Figs 6 and 7 present the effects of wall mass fractions on the velocity profiles. It is noted that velocity profile *f*′ shows opposite behavior for the first and second solutions. Crossover and oscillations are also observed for the suction parameter S in the second solution profile. Again we can say that both are real solutions as the first predicts the decrease in magnitude of velocity particles whereas the second gives the crossover behavior. The influence of heat source (*λ*_1_ > 0) parameter on temperature profile *θ* is elucidated in the Fig 8. It is noted that heat source enhance the thermal conductivity and increases the fluids’ temperature. Fig 9 incorporates the effects of heat sink parameter (*λ*_1_ < 0). As expected heat sink provides a decrease in the temperature of fluid. In order to discuss the results of local Nusselt number $Nu_{x}/Re_{x}^{1/2}$ against different physical quantities including Deborah number, Prandtl number and internal heat generation/absorption quantity, we have prepared Table 1. It is evident from this table that an increase in Deborah number and internal heat generation/absorption quantity, the local Nusselt number decreases whereas due to an increase in Prandtl number, the local Nusselt number increases. Table 2 presents the nomenclature.

**Table 1:** Results of Local Nusselt number −*θ*′(0) for different physical quantities including Deborah number *β*, Prandtl number Pr, internal heat generation/absorption parameter *λ*_1_.

*β*	Pr	*λ*_1_	−*θ*′(0)
0.0	1.0	0.1	2.1475
0.2			2.0068
0.4			1.7395
0.2	0.1		0.3268
	0.5		0.9806
	1.2		2.4130
	2.0		1.2797
	1.0	-0.4	2.2309
		-0.2	2.1452
		0.0	2.0554
		0.2	1.9531
		0.4	1.8401

**Table 2:** Nomenclature.

*u, v*	Velocity components (ms^-1^)	*Greeks symbol*	
*x, y*	Cartesian coordinates (l)	*λ*	Relaxation time (t)
*U*_*w*_, *V*_*w*_	Wall velocities (ms^-1^)	*ν*	Kinematic viscosity (m^2^s^-1^)
*C*	Dimensional constant (t^-1^)	*ρ*	Density of fluid (kgm^-3^)
*T*	Fluids’ temperature (K)	*λ*_1_	Internal heat generation/absorption parameter
*T*_*w*_	Wall temperature (K)	*σ*	Electrical conductivity parameter
*T*_∞_	Ambient temperature (K)	*ϕ*	Stream function
*M*	Magnetic field (Te)	*η*	Similarity variable
*S*	Suctions/ injection parameter (ms^-1^)	*β*	Deborah number
*Q*	Heat source/sink parameter	*θ*	Dimensionless temperature
Pr	Prandtl number		
*Nu*_*x*_	Nusselt number		
*f*	similarity variable		
*k*	Thermal conductivity		

## Conclusions

This study presents the influence of internal heat generation/absorption in a nonlinear fluid flow over a permeable wall. The dynamics of the magneto-hydrodynamic fluid flow in porous medium over a porous wall are investigated. The final outcomes of the presented analysis are listed below:

- Dual solutions exists for the velocity and temperature profiles for the shrinking wall.
- For stretching geometry only unique solutions exist for velocity and temperature.
- For the permeability parameter, the second solution is found to be dominant when compared with the first solution.
- The effects of suction for the first and second solution are quite opposite. First solution predicts the decrease in magnitude of velocity particles whereas the second gives the crossover behavior.
- The influence of heat source (*λ*_1_ > 0) parameter on *θ* give an increase in the temperature of fluid while heat sink parameter (*λ*_1_ < 0) provides a decrease in the temperature of fluid.
